# A Nomogram for Predicting BK Virus Activation in Kidney Transplantation Recipients Using Clinical Risk Factors

**DOI:** 10.3389/fmed.2022.770699

**Published:** 2022-02-10

**Authors:** Jiyan Wang, Jiawei Li, Zhongli Chen, Ming Xu, Cheng Yang, Ruiming Rong, Tongyu Zhu

**Affiliations:** ^1^Department of Urology, Zhongshan Hospital, Fudan University, Shanghai, China; ^2^Shanghai Key Laboratory of Organ Transplantation, Shanghai, China; ^3^State Key Laboratory of Cardiovascular Disease, Fuwai Hospital, National Center for Cardiovascular Diseases, Chinese Academy of Medical Sciences and Peking Union Medical College, Beijing, China; ^4^Zhangjiang Institute of Fudan University, Shanghai, China; ^5^Department of Transfusion, Zhongshan Hospital, Fudan University, Shanghai, China

**Keywords:** BK virus, kidney transplantation, risk factor, predictive model, nomogram

## Abstract

BK virus is a common opportunistic viral infection that could cause BK virus-associated nephropathy in renal transplant recipients. Thus, we retrospectively analyzed clinical and laboratory data associated with a higher risk of BK virus activation from 195 renal transplant recipients by the multivariate logistic regression analysis and performed the external validation. Results showed that patients with BK virus active infection were associated with a deceased donor, had lower direct bilirubin levels, a higher proportion of albumin in serum protein electrophoresis, and lower red blood cells and neutrophil counts. The multivariate logistic regression analyses revealed that the living donor, direct bilirubin, and neutrophil counts were significantly associated with BK virus activation. The logistic regression model displayed a modest discriminability with the area under the receiver operating characteristic curve of 0.689 (95% CI: 0.607–0.771; *P* < 0.01) and also demonstrated a good performance in the external validation dataset (the area under the receiver operating characteristic curve was 0.699, 95% CI: 0.5899–0.8081). The novel predictive nomogram achieved a good prediction of BK virus activation in kidney transplant recipients.

## Introduction

BK virus (BKV) is a common post-transplant opportunistic viral infection that can cause interstitial nephritis and allograft failure in renal transplant recipients (RTRs) (1, 2). With the progress of the immunosuppressive regimen, acute rejection incidence decreased. However, viral infections after renal transplantation are still a hurdle, which causes chronic allograft loss. BKV is one member of the polyomavirus family, which was first described in 1971 in the urine of an RTR with ureteric stenosis. BKV has circular nucleic acid and double-stranded DNA (3). The transmission mechanism of BKV is unclear but supposed through the mouth and respiratory tract (4).

Primary infection of BKV, such as intermittent asymptomatic viral shedding in the urine, can be detected in healthy individuals with no adverse outcome. However, in the immunosuppressed status, BKV will be reactivated, leading to clinically BKV disease among the renal and hematopoietic stem cell recipients (5). After being activated in the early stage of BK virus-associated nephropathy (BKPyVN), BKV in the urothelium and tubular epithelium will replicate in high levels. Some virus compositions, such as BKV-DNA and decoy cells, can be detected in urine, diagnosed as BKV viruria. As the damage escalates, the BKV virus will invade into circulation through capillary, causing the BKV viremia. According to previous studies, the prevalence of BK viruria, BK viremia, and BKPyVN range from 10 to 40% in RTRs, and this progressively affects graft function and increases the risk of graft loss more than 10% (4, 6–9). Besides, BKV was also associated with *de-novo* donor-specific antibodies, which were linked to antibody-mediated rejection (10). In addition, the association between BKV and malignancy has attracted more attention. Some kinds of human neoplasms such as urothelial bladder cancer can detect BKV-DNA sequences and T antigen (11–13).

Detection of BKV-DNA load in urine and plasma by PCR is a common and effective way to diagnose and monitor BKPyVN and monitor BKPyVN at the early stage. Positive BKV-DNA in urine or plasma has a good negative predictive value, but the positive predictive value is unsatisfactory (14). Current investigations showed that RTRs with a BKV-DNA load of more than 1.0 × 10^7^ copies/ml in urine or more than 1 × 10^4^ copies/ml in plasma have a higher risk of developing into BKPyVN (15–17). This state is considered as BKV activation. This DNA level is also found linked to hemorrhagic cystitis (18–20). Some researchers suggested that the viral replication capacity is associated with rearranged-noncoding control regions (21). Unfortunately, effective BK virus–specific antiviral therapies are not available. Therefore, early diagnosis of BKV activation and intervention is of great clinical importance (22, 23).

In this study, we analyzed the clinical and laboratory data among 195 RTRs to build a predictive model for BKV active replication.

## Methods

### Patients

**Figure 1:**
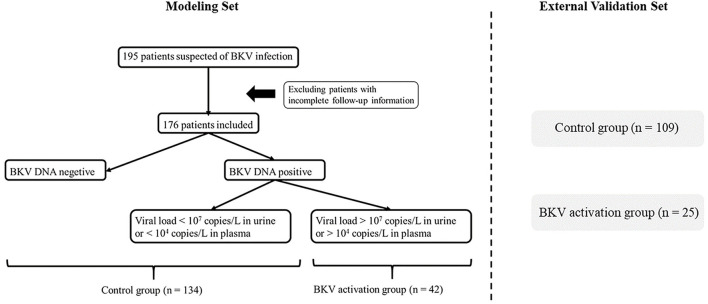
Flowchart of patient enrollment. After deleting invalid cases through the exclusion criteria, 176 patients were enrolled and divided into the BK virus (BKV) activation group (*n* = 42) and the control group (*n* = 134) according to BKV-DNA levels in plasma and urine.

In the training cohort, we selected 196 patients undergoing renal transplantation in Zhongshan Hospital, Fudan University from February 2018 to April 2020. Only RTRs who accepted complete follow-up laboratory examinations and BKV-DNA testing in plasma and urine were included. Inclusion criteria were: living or deceased donor kidney transplant and recipient age ≥ 18 years (24). Exclusion criteria included recipients with primary immune dysfunction, acute and central nervous system diseases, hemolytic anemia, coagulation dysfunction, HIV and other high-risk pathogens infection, pregnant or lactating women, and other conditions that the investigators deemed inappropriate for participation during the study period. After all, the demographic and clinical information data were collected, 176 RTRs were included (Figure 1). All the patients were divided into two groups according to BKV-DNA levels in plasma and urine. The BKV activation group was defined as BKV-DNA > 10^7^ copies/L in the urine or >10^4^ copies/L in the plasma (*n* = 42). Others were defined as the BKV inactivation group (*n* = 134). The external cohort with 134 RTRs was enrolled from May 2020 to Oct 2021 in Zhongshan Hospital, Fudan University.

This study was approved by the Institutional Review Board (IRB) at Zhongshan Hospital, Fudan University, Shanghai, China (approval. No.: B2021-074R) and registered and recorded at the Human Genetic Resource Administration of China (HGRAC) (reference no.: 2021BAT1438).

### Data Collection

The demographic features (gender and age), disease history (diabetes, coronary heart disease, hypertension, transfusion, malignancy, hyperlipemia, and viral hepatitis history), and transplantation history (source of donor, transplant number, ABO compatibility, ischemia time, acute rejection or delayed graft function history, induction treatment, and initial immunosuppressive protocol) were collected by two clinicians. The post-transplantation laboratory tests [blood routine included: red blood cells (RBCs) count, platelets count, neutrophils count, lymphocyte count, and monocyte count; liver function including alanine aminotransaminase, aspartate aminotransferase, total bilirubin, and direct bilirubin (DB); renal function included serum creatine, urea nitrogen, estimated glomerular filtration rate (eGFR), and uric acid; the proportion of serum protein electrophoresis (SPE); and urine protein semiquantitative levels] were also collected at the same time of BKV-DNA test.

### Statistical Analysis

For continuous variables, the one fitting normal distribution is expressed as the mean ± SD, otherwise described as the median with interquartile ranges. For categorical data, the proportions and frequencies are calculated. Continuous and categorical variables were compared using the independent *t*-test or nonparametric and the chi-squared tests, respectively. The obvious abnormal distribution variables were normalized by natural logarithmic transformation. Spearman's correlation analyses were adopted to explore the relationship between variables.

The univariate analysis was performed to screen the potential variables associated with BKV activation. Variables with *P*-value < 0.1 in the univariate analysis (living-related donor, DB, globulin, SPE-albumin, SPE-β, RBC, platelet, and neutrophil counts (as a natural logarithm-transformed continuous variable, represented as “ln Neutr”)], demographic features, and clinically important factors such as serum creatinine were further included in the multivariate logistic regression analysis (25). Using the forward stepwise method, the final logistic regression model was constructed. Odds ratios (ORs) and 95% CIs were presented. For clinical usage, a nomogram was constructed based on the model. The goodness-of-fit was estimated by the Hosmer–Lemeshow test and discriminability was assessed by the area under the receiver operating characteristic (AUROC) curve.

The performance of the model was also evaluated in the different age and sex groups as well as an external validation cohort. All the statistical analyses were performed using SPSS software, version 20.0 (SPSS Incorporation, Chicago, Illinois, USA) and R software version 3.6.3. Statistical significance was set at two-tailed and *p* < 0.05 was considered as statistically significant. All the authors had full access to the data in this study and took responsibility for data integrity and accuracy of data analysis.

## Results

### Characteristics of Patients

The variables included age, gender, the history of diabetes, coronary heart disease, hypertension, transfusion, malignancy, hyperlipemia, viral hepatitis, the information of donor source, transplantation times, ABO compatible, ischemia time, acute rejection or delayed graft function history, induction treatment, initial immunosuppressive protocol (include the combined regimen of tacrolimus, mycophenolic acid, and prednisone, represented as Tac + MPA + Pred and the combined regimen of cyclosporin A, mycophenolic acid, and prednisone, represented as CsA + MPA + Pred), and BKV-DNA quantitative results in plasma and urine. Other routine follow-up laboratory results were also included: RBC, platelet, neutrophil count, lymphocyte count, monocyte count, total bilirubin, DB, globulin, the proportion of SPE, alanine aminotransferase, aspartate aminotransferase, creatine, urea nitrogen, uric acid, estimated glomerular filtration rate (eGFR), and urine protein semiquantitative levels.

The clinical characteristics are shown in Table 1. There are 65 patients with BKV-DNA positive in urine or plasma and 42 of them were in virus activation according to virus load. When comparing the control group (*n* = 134) and the BKV activation group (*n* = 42), living donor proportion (28.4 vs. 9.5%), DB [2.9 (1.7, 4.0) vs. 2.4 (1.6, 2.8) μmol/l], SPE-Albumin (61.7 ± 5.0 vs. 63.5 ± 4.2%), and RBC count (3.90 ± 0.83 × 10^12^/L vs. 3.52 ± 0.72 × 10^12^/L) showed significant difference. The two groups did not differ with respect to gender, age, the history of diabetes, coronary heart disease, hypertension, transfusion, malignancy, hyperlipemia, viral hepatitis, the information of transplantation times, ABO compatible, ischemia time, acute rejection or delayed graft function history, induction treatment, initial immunosuppressive protocol, the levels of total bilirubin, total protein, globulin, alanine aminotransferase, aspartate aminotransferase, creatine, urea nitrogen, uric acid, eGFR in serum, the proportion of serum α1, α2, β, γ protein in SPE, the count of platelet, neutrophils, lymphocytes, and monocytes in peripheral blood and urine protein semiquantitative levels.

**Table 1:** Baseline characteristics for patients with or without BKV active replication.

	**All**	**Inactivated**	**Activated**	**P-value**
	**(*n =* 176)**	**(*n =* 134)**	**(*n =* 42)**	
**Sex**				0.669
Male	122 (69.3)	94 (70.1)	28 (66.7)	
Female	54 (30.7)	40 (29.9)	14 (33.3)	
**Age**	43.0 ± 12	42.2 ± 12.1	45.4 ± 11.8	0.139
**Diabetes**	30 (17.0)	24 (17.9)	6 (14.3)	0.586
**Coronary heart disease**	2 (1.1)	2 (1.5)	0 (0)	0.579
**Hypertension**	150 (85.2)	118 (88.1)	41 (97.6)	0.126
**Transfusion**	19 (10.8)	14 (10.4)	5 (11.9)	1.000
**Malignancy**	4 (2.3)	3 (2.2)	3 (7.1)	0.744
**Hyperlipemia**	21 (11.9)	15 (11.2)	6 (14.3)	0.590
**Hepatitis**				
HBV	26 (14.8)	16 (11.9)	10 (23.8)	0.059
HCV	3 (1.7)	3 (2.2)	0 (0)	1.000
**First time transplant**	168 (95.5)	128 (95.5)	40 (95.2)	1.000
**Donor source**				0.012
Living donor	42 (23.9)	38 (28.4)	4 (9.5)	
Deseased donor	134 (76.1)	96 (71.6)	38 (90.5)	
**ABO-compatible**	173 (98.3)	132 (98.5)	41 (97.6)	0.699
**Ischemia time**				
Cold ischemia time (hour)	5.1 (1.0, 9.5)	5.0 (0.9, 9.5)	5.1 (0.9, 10)	0.863
Warm ischemia time (min)	2.7 (2.0, 3.0)	2.6 (2.0, 3.0)	2.7 (2.0, 3.0)	0.470
**Acute rejection**	2 (1.1)	0	2 (4.8)	0.056
**Delayed graft function**	14 (8.0)	12 (9.0)	2 (4.8)	0.583
**Intravenous immunogloblin**	2 (1.1)	2 (1.5)	0 (0)	1.000
**Induction treatment**				1.000
Antithymocyte globulin	8 (4.5)	6 (4.5)	2 (4.8)	
Basiliximab	168 (95.5)	128 (95.5)	40 (95.2)	
**Initial immunosuppressive protocol**				0.171
Tac+MPA+Pred	112 (63.6)	89 (66.4)	23 (54.8)	
CsA+MPA+Pred	64 (36.4)	45 (33.6)	19 (45.2)	
**Kidney and liver function**				
Total bilirubin (μmol/L)	8.0 (5.8, 10.4)	8.2 (5.8, 11.3)	7.5 (5.7, 9.0)	0.139
DB (μmol/L)	2.6 (1.7, 3.5)	2.9 (1.7, 4.0)	2.4 (1.6, 2.8)	0.012
Total protein (g/L)	65 ± 7	66 ± 8	64 ± 6	0.047
Albumin (g/L)	42 ± 5	43 ± 5	42 ± 3	0.247
Globulin (g/L)	22 ± 4	23 ± 4	22 ± 4	0.066
Alanine aminotransferase (U/L)	15 (9, 27)	15 (9, 26)	14 (9, 29)	0.804
Aspartic aminotransferase (U/L)	16 (13, 21)	16 (13, 21)	15 (11, 21)	0.465
Creatine (μmol/L)	152 (113, 196)	152 (113, 189)	154 (116, 272)	0.497
Urea nitrogen (mmol/L)	9.2 (7.2, 12.8)	9.2 (7.2, 12.7)	9.3 (7.2, 13.8)	0.569
Uric acid (μmol/L)	387 ± 113	390 ± 115	376 ± 106	0.480
eGFR (ml/min/1.73m^2^)	47 ± 23	48 ± 22	43 ± 24	0.223
**Serum Protein electrophoresis**				
Albumin (%)	62.2 ± 4.9	61.7 ± 5.0	63.5 ± 4.2	0.043
α1 (%)	4.8 ± 1.2	4.8 ± 1.3	4.6 ± 1.1	0.524
α2 (%)	9.7 ± 2.3	9.8 ± 2.3	9.3 ± 2.0	0.214
β (%)	10.3 ± 1.4	10.4 ± 1.4	9.9 ± 1.2	0.074
γ (%)	13.1 ± 3.3	13.2 ± 3.3	12.6 ± 3.3	0.265
**Blood count**				
RBC (×10^12^/L)	3.81 ± 0.82	3.90 ± 0.83	3.52 ± 0.72	0.008
PLT/10 (×10^9^/L)	19.9 ± 6.6	20.4 ± 6.9	18.3 ± 5.3	0.072
Neutrophil (×10^9^/L)	4.7 (3.6, 6.3)	5.0 (3.7, 64)	4.3 (3.1, 5.8)	0.367
Lymphocyte (×10^9^/L)	1.6 ± 0.8	1.6 ± 0.8	1.4 ± 0.7	0.200
Monocyte (×10^9^/L)	0.58 ± 0.24	0.58 ± 0.24	0.59 ± 0.22	0.940
**Urine protein**				0.121
(-)	112 (63.7)	85 (63.4)	27 (64.3)	
(±)	36 (20.5)	28 (20.9)	8 (19.0)	
(+~++)	16 (9.1)	9 (6.7)	7 (16.7)	
(++~+++)	9 (5.1)	9 (6.7)	/	
(+++~++++)	3 (1.7)	3 (2.2)	/	

*For continuous variables, the fitting normal distribution ones are expressed as the mean ± SD, otherwise expressed as the median with interquartile ranges for data. For categorical data, the proportions and frequencies are calculated. Continuous and categorical variables were compared using the independent t-test or the nonparametric and chi-squared tests*.

*BKV, BK virus; DB, direct bilirubin; Tac, tacrolimus; CsA, cyclosporine A; MPA, mycophenolic acid; Pre, prednisone; eGFR, estimated glomerular filtration rate; RBC, red blood cell*.

In the external validation cohort, patients (*n* = 134) were among middle-aged (46 ± 12 years) and 64.9% were men. A total of 25 patients were divided into the BKV activation group. The clinical characteristics are shown in Supplementary Table S1. Comparing the two groups, neutrophil count [5.3 (3.4, 7.1) × 10^9^ vs. 4.3 (3.2, 5.4) × 10^9^] showed a significant statistical difference. The two groups did not differ with respect to gender, age, the history of diabetes, coronary heart disease, hypertension, transfusion, malignancy, hyperlipemia, viral hepatitis, the information of transplantation times, ABO compatible, ischemia time, acute rejection or delayed graft function history, induction treatment, initial immunosuppressive protocol, the levels of total bilirubin, DB, total protein, albumin, globulin, alanine aminotransferase, aspartate aminotransferase, creatine, urea nitrogen, uric acid, eGFR in serum, the proportion of serum α1, α2, β, γ protein in SPE, the count of RBC, platelet, neutrophils, lymphocytes, and monocytes in peripheral blood and urine protein semiquantitative levels.

### Clinical Factors Associated With BKV Activation

The univariate analyses showed that BKV activation was associated with living donors, DB (as a natural logarithm-transformed continuous variable, represented as “ln DB”), globulin, SPE-β, RBC, platelet, and neutrophil counts (as a natural logarithm-transformed continuous variable, represented as “ln Neutr”) (Table 2). In the multivariate logistic regression analyses, the living donor, DB level, and neutrophil count were selected as significant variables in the model. Specifically, for patients receiving the kidney graft from a living donor, there was a 74% lower likelihood of having BKV activation status. Among patients in our study, higher levels of DB and neutrophil counts were associated with lower odds of BKV activation.

**Table 2:** The multivariate logistic regression models for renal transplant recipients (RTRs) with BKV activation.

	**Univariate analysis**		**Multivariable analysis**	
	**OR**	**P-value**	**OR**	**P-value**
Living related donor	0.266	0.018	0.260	0.019
Ln DB	0.515	0.034	0.501	0.038
Globulin	0.923	0.068	–	–
SPE-Albumin	1.086	0.046	–	–
SPE-β	0.784	0.076	–	–
RBC	0.563	0.010	–	–
Platelet	0.995	0.075	–	–
Ln Neutr	0.492	0.035	0.487	0.053

*Age, gender, living-related donor, ln DB, globulin, serum protein electrophoresis (SPE)-Albumin, SPE-β, red blood cell (RBC) count, platelet count, and ln Neutr were included*.

### Evaluation of Model Performance

**Figure 2:**
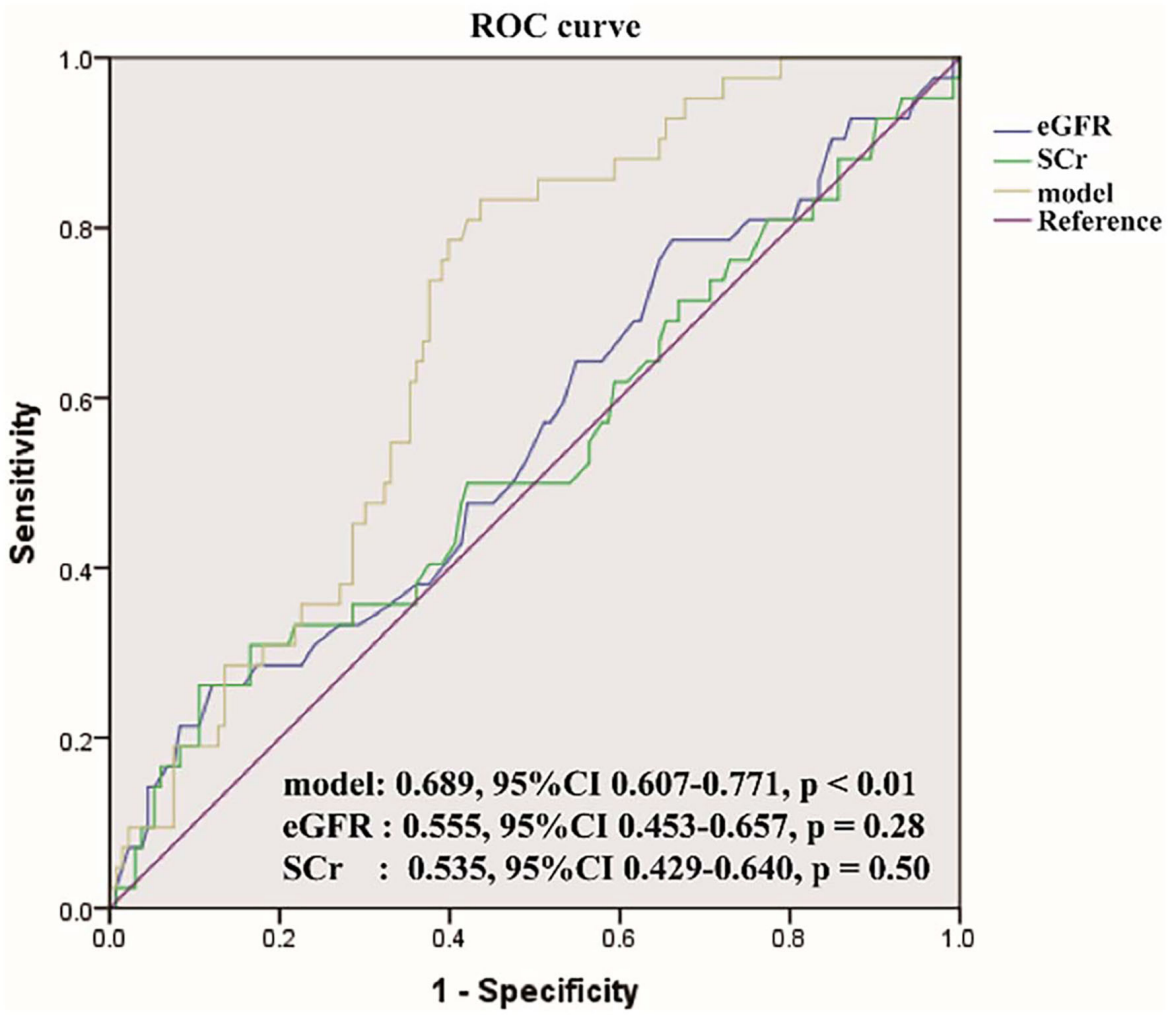
The receiver operating characteristic (ROC) curve of the model compared with serum creatinine (SCr) and estimated glomerular filtration rate (eGFR). The area under the ROC (AUROC) of the three models was 0.689, 0.555, 0.535, respectively, indicating that model has better predictive power.

**Figure 3:**
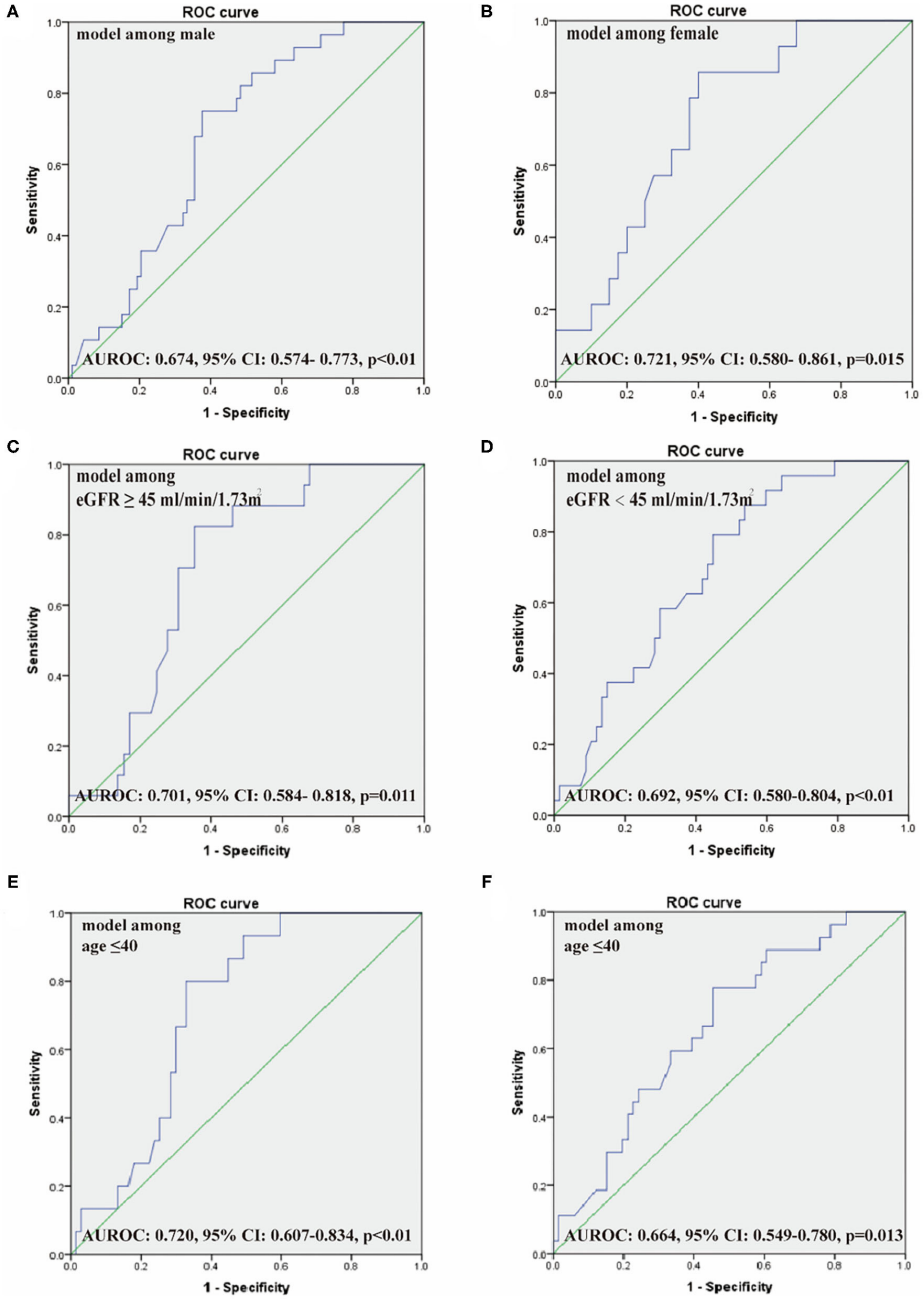
The ROC of our model among male **(A)** and female patients **(B)**, eGFR ≥ 45 ml/min/1.73 m^2^ **(C)** and <45 ml/min/1.73 m^2^ **(D)**, age ≤ 40 years **(E)** and > 40 years **(F)**. AUROC, the area under the receiver operating characteristic curve.

**Figure 4:**
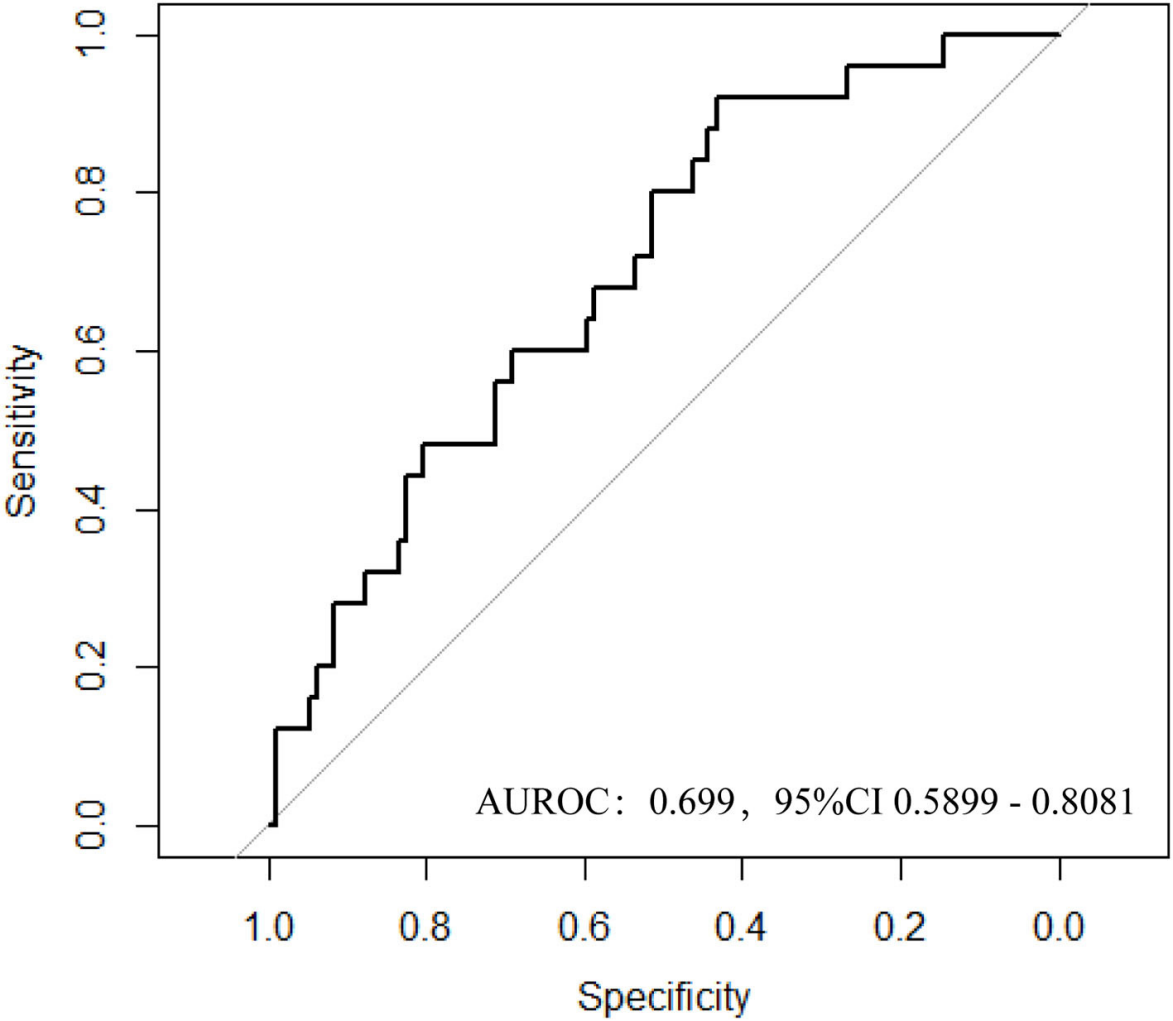
Discriminatory performance in external datasets for adverse BKV infection status.

The logistic regression model [P/(1-P) = exp [0.745–1.347 (living donor)−0.691 (ln DB)−0.720 (ln Neutr)] displayed a good fit with a Nagelkerke R^2^ value of 0.143 and the nonsignificant Hosmer–Lemeshow test. The ROC analysis showed that the AUROC of the model was 0.689 (95% CI: 0.607–0.771; *P* < 0.01) for distinguishing the presence of BKV activation (Figure 2), which was better than the creatine or eGFR. To test the robustness of the model, we applied the model to the different age, sex, or eGFR subgroups and an independent cohort. Notably, the model demonstrated higher discriminability in female (AUROC, 0.721 vs. male 0.674) and younger patients (AUROC, 0.720 vs. male 0.664) (Figure 3), while its AUROC was roughly equal between the eGFR ≥ 45 ml/min/1.73 m^2^ and < 45 ml/min/1.73 m^2^ groups. Additionally, the model retained modest discrimination in the external validation dataset, although the AUROC slightly decreased (0.699, 95% CI: 0.5899–0.8081) (Figure 4).

### Predictive Nomogram for the Probability of BKV Reactivation

**Figure 5:**
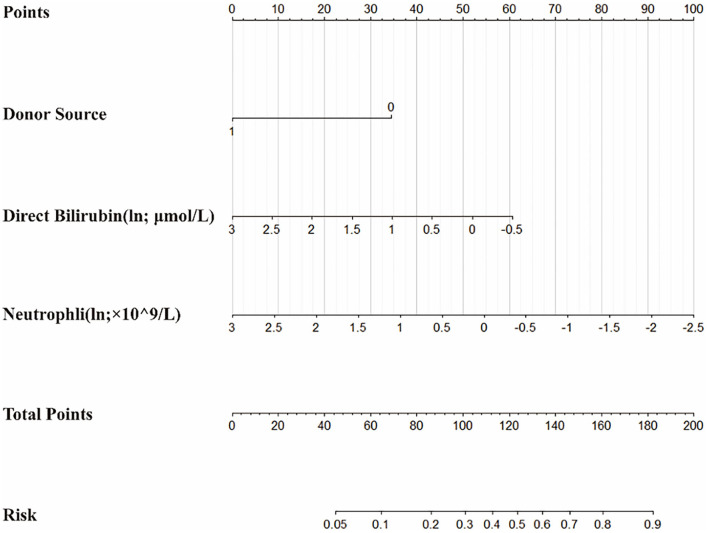
The nomogram to estimate the risk of BKV activation in renal transplant recipients (RTRs). Based on the nomogram, the position of each variable on the corresponding axis can match a point to the points axis. The sum points of all the variables can draw a line from the total points axis to the risk axis and obtain the probabilities.

On the basis of the multivariate logistic regression model, a nomogram was constructed for predicting BKV activation (Figure 5). A total score was calculated using donor source, ln DB and ln Neutr. The value of each of these variables on the corresponding can match a number to a score on the point scale axis. A sum of all the variable scores could draw a line to the total point axis and acquire the probability of BKV activation.

## Discussion

BK virus infection is a common post-transplant opportunistic infection. The relationship between BKV and renal allograft dysfunction is still unclear and the effective management strategies remain to warrant further investigation. In the view of the facts that are more relevant between BKV activation and graft loss and the few effective BK virus-specific treatment, identifying BKV activation patients as early as possible to increase post-transplant surveillance and promptly adjust treatments are of greater clinical value. In this article, we compared the clinical data and laboratory results among RTRs to build a prediction model for BKV active replication.

Previous studies had come up with many but controversial risk factors of BKV infection, including age, gender, human leukocyte antigen (HLA) mismatches, deceased donor transplants, duration of cold ischemic time, body mass index, and types of immunosuppressive drugs (26–28). All of these have yet to be widespread corroborated. In a multi-center retrospective study including 21,575 mate kidney transplant pairs, age <18 or ≥60 years, male sex, depleting antibody, HLA mismatch ≥ 4 were identified as the risk factors of BKV (27). The results showed that age, gender, HLA mismatches, acute rejection, and the use of depleting antibody induction of recipients were associated with a higher odd value of BKV treatment. Another retrospective analysis for identification of BKV infection among living-donor RTRs showed that tacrolimus level and decreased lymphocyte percentage might be the risk factors (29). The results vary from research to research, which may be related to the different clinical manifestations between BKV infection and damage.

In our study, an effective predictive model for identifying BKV activation was established by the multivariate logistic regression analyses. In this model, the living donor, tDB level, and neutrophil count were found that have significant differences for predicting the presence of BKV activation. In verifying the model performance, the ROC and the AUROC of the model showed better performance than routine renal function index [included serum creatinine (SCr) and eGFR]. Also, after comparing the ROC of our model among male and female patients, eGFR ≥ 45 ml/min/1.73 m^2^ and <45 ml/min/1.73 m^2^, and age ≤ 40 years and >40 years, this model showed more power in female and younger patients. For visualization, a nomogram was constructed by incorporating the three significant factors. In further validating the model efficiency, the predictive ability of the model in external validation shown as the ROC still had a certain power.

The common diagnostic measures of BKV include seeking decoy cells in urinary sediment, test virus DNA in urine or plasma by PCR, or graft biopsy. All the measurements have different merits and demerits. The sensitivity of urinary sediment cytology is poor and the positive results often along with the high level of virus replication miss the early. The biopsy is a gold standard for diagnosis of BKPyVN by immunohistochemistry staining SV40 and LT antigen, but it is also an invasive detection and possible to be false positive due to the bias of puncture position. Detection of the viral load in urine and plasma by PCR is an effective way to monitor diseases in the early stage. The levels of BKV-DNA in urine and plasma are highly correlated with BKPyVN. However, positive BKV-DNA in urine or plasma has a good negative predictive value, but an unsatisfactory positive predictive value (14). It is highly sensitive for reminding active viral replication but not for BKPyVN. Current foreign and domestic investigations showed that people whose BKV-DNA load is more than 1.0 × 107 copies/ml in urine and more than 1 × 104 copies/ml in plasma have a higher risk of developing into BKPyVN and need timely intervention (15–17). Meanwhile, there are no effective BK virus-specific antiviral therapies available (22, 23). The mainstay for managing reactivation still relies on regular screening accompanied by a reduction of immunosuppressant and adding antiviral drugs, if the former is in vain (30). However, this approach proves effective only in 50–80% of cases and does not eliminate the potential evolution to BKPyVN (30). Therefore, identifying this kind of patient as early as possible and timely intervention to improve patients' prognosis are of great significance.

The source of the donor was screened as an independent protective factor of BKV activation, which means that RTRs whose graft from living donors are less likely to BKV reactivation. This is consistent with some previous studies. A systematic review and meta-analysis of risk factors for BKV viremia and BKPyVAN showed that deceased donor is one of the risk factors (31). In the included multivariate analyses studies, the whole six articles included showed strong associations between BKPyV viremia and deceased donor condition and the OR value of two of them has statistical differences (32, 33). However, there is no clear theoretical basis supporting the correlation between the source of graft and BKV activation. Clinical data seem to only provide this phenomenon but a clue. It can be supposed that the virus activation may not only relate to the recipient condition but also the donor condition.

As a normal marker responding to hepatocyte metabolism, most of DB is derived from the breakdown of hemoglobin (Hb) in erythrocytes and formulated in the liver (34). Even though a little study of BKV and DB can be retrieved, serum DB levels have been always attended in the clinical studies of other virus-associated diseases. For example, a retrospective study of patients with COVID-19 in Guangzhou found that DB was one of the independent risk factors for the occurrence of critical illness (35). In this study, serum DB levels seem like a predictor factor. The antiviral effect of DB has little clue in current literature, but it was mentioned in hepatitis A virus (HAV) infection. DB may differentially regulate CD4+ T lymphocytes and T-regulatory cells functions by modulating intracellular pathways and cellular receptor expression to affect T-cell function during HAV infection (36). Another study indicated that DB may affect cytokine profiles in HAV infection by modulating signal transducers and activators of transcription proteins acting as a potential immunomodulator (37). Moreover, its protective function may be associated with antioxidation. Some studies suggested that bilirubin, at micromolar concentrations in vitro, efficiently scavenges peroxyl radicals generated chemically in either homogeneous solution or multilamellar liposomes (38). There are other studies in hepatitis virus that found the antioxidant properties and immunomodulation function of DB (37, 39). Therefore, combining the basis that BKV reactivation and causing related diseases are associated with T lymphocytes, DB may also play an antiviral effect in BKV activation by modulating T lymphocytes function.

Neutrophils in peripheral blood are the main components of white blood cells and a common indicator, which can generally reflect body immunologic function. BKV infection has been associated with hemorrhagic cystitis in recipients of hematopoietic stem cell transplants, suggesting that reactivation of this infection might have some relationship with diminished immune surveillance (15). Previous studies strongly supported the occurrence of BKV-associated graft loss or cancer associated with BKV reactivation due to the low immunity (4, 9). In our study, the results that neutrophil count was regarded as a protective factor in BKV activation can be simply explained as mapping the capacity for resisting BKV reactivation. Otherwise, many studies pointed out the correlation between lymphocytes count and BKV, even though the lymphocytes count did not perform obvious results in our analyses. The lymphocyte count was reportedly lower in patients with BKV viremia than in patients with no viremia (40, 41). It is unknown whether different parts of leukocytes play a respective role in BKV infection and how various phenotypes lymphocytes regulate the BKV reactivation pathological process.

In our result, the AUROC is different between men and women. One of the reasons may be the original different proportions of kidney transplant recipients between men and women whether of BKV activation. The number of included female cases was less than male in the same period. The statistical result reflected that the power in women was not necessarily accurate. Another reason may be the genetic heterogeneity between the X and Y chromosomes leading to sex differences persisting throughout the whole body and life (42). The different power between men and women is possibly objective existence. Further research including more cases in multicenter is needed to explore the model power between both the gender and possible mechanism if the difference exists.

There are some aspects of this study that should be noted as limitations. Our model was a single-center study for 2 years so the limited number of cases included impact on the model power. In addition, the results of performance evaluation showed that the prediction ability of the model is superior to normal renal function but is not excellent. The reason may go back to the included variable, which suggested that high specificity and correlation indexes are waiting for application into clinical practice.

## Conclusion

This study suggested several clinical factors associated with BKV activation after renal transplantation and we built a model for identifying BKV status. The potential clinical implications for taking advantage of routine follow-up laboratory examines to monitor and predict BKV infection and activation still need to go further.

## Data Availability Statement

The datasets presented in this study can be found in online repositories. The names of the repository/repositories and accession number(s) can be found below: the Human Genetic Resource Administration of China, https://fuwu.most.gov.cn/, 2021BAT1438.

## Ethics Statement

This study was approved by the Institutional Review Board (IRB) at Zhongshan Hospital, Fudan University, Shanghai, China (Approval. No.: B2021-074R) and registered and recorded at the Human Genetic Resource Administration of China (HGRAC) (Reference No.: 2021BAT1438).

## Author Contributions

TZ, CY, and RR contributed to the conceptualization, methodology, funding acquisition, and project administration. JW, JL, and ZC contributed to the formal analysis, data curation, investigation, and writing review and editing. JW and JL contributed to the writing the original draft preparation. MX contributed to the supervision. All the authors have read and agreed to the published version of the manuscript.

## Funding

This study was supported by the National Key R&D Program of China (2018YFA0107501 to RR, 2018YFA0107502 to CY), the National Natural Science Foundation of China (81770746 to CY, 81770747 and 81970646 to RR), the Shanghai Rising Star Program (19QA1406300 to CY), the Medical and Health Talents Training Plan for the Excellent Youth of Shanghai Municipal (2018YQ50 to CY), the 2019 Shanghai Youth Talent Development Program (to CY), the Science and Technology Commission of Shanghai Municipality (16431902300 to TZ), and the Zhongshan Hospital of China (SYS-054 to TZ).

## Conflict of Interest

The authors declare that the research was conducted in the absence of any commercial or financial relationships that could be construed as a potential conflict of interest.

## Publisher's Note

All claims expressed in this article are solely those of the authors and do not necessarily represent those of their affiliated organizations, or those of the publisher, the editors and the reviewers. Any product that may be evaluated in this article, or claim that may be made by its manufacturer, is not guaranteed or endorsed by the publisher.

## Supplementary Material

Supplementary Table 1S1 The clinical characteristics of external validation patients.

## Abbreviations

BKPyVAN: BK virus-associated nephropathy
BKV: BK virus
DB: direct bilirubin
eGFR: estimated glomerular filtration rate
OR: Odds Ratios
PCR: polymerase chain reaction
RBC: red blood cells
ROC: Receiver Operating Characteristic Curve
AUROC: the area under ROC
RTRs: renal transplant recipients
SPE: serum protein electrophoresis.
