# Supplementary material for: A Nomogram for Predicting BK Virus Activation in Kidney Transplantation Recipients Using Clinical Risk Factors
Source: Front Med (Lausanne). 2022 Feb 10;9:770699. doi: 10.3389/fmed.2022.770699 (PMC8866320; doi:10.3389/fmed.2022.770699)
Supplement: Supplementary Table 1S1 — The clinical characteristics of external validation patients. [file Table_1.docx]

**Supplement Table 1 Clinical characteristics of external validation patients**

|  |  | **All** | **Inactivated** | **Activated** | **P value** |
| --- | --- | --- | --- | --- | --- |
|  |  | **(n = 134)** | **(n=109)** | **(n=25)** |  |
| **Sex** | |  |  |  | 0.133 |
|  | Male | 87 (64.9) | 74 (67.9) | 13 (52.0) |  |
|  | Female | 47 (35.1) | 35 (32.1) | 12 (48.0) |  |
| **Age** | | 46 ± 12 | 46 ± 13 | 46 ± 11 | 0.945 |
| **Diabetes** | | 12 (9.0) | 10 (9.2) | 2 (8.0) | 1.000 |
| **Coronary heart disease** | | 1 (0.7) | 1 (0.9) | 0 (0) | 1.000 |
| **Hypertension** | | 114 (85.1) | 94 (86.2) | 20 (80.0) | 0.335 |
| **Transfusion** | | 23 (17.2) | 19 (17.4) | 4 (16.0) | 1.000 |
| **Malignancy** | | 3 (2.2) | 2 (1.8) | 1 (4.0) | 0.465 |
| **Hyperlipemia** |  | 8 (5.9) | 6 (5.5) | 2 (8.0) | 0.994 |
| **Hepatitis** |  |  |  |  |  |
|  | HBV | 7 (5.2) | 7 (6.4) | 0 (0.0) | 0.422 |
|  | HCV | 1 (0.7) | 1 (0.9) | 0 (0.0) | 1.000 |
| **First time transplant** | | 131 (97.8) | 106 (97.2) | 25 (100.0) | 1.000 |
| **Donor source** |  |  |  |  | 0.488 |
|  | Living donor | 29 (21.6) | 25 (22.9) | 4 (16.0) |  |
|  | Deseased donor | 105 (78.4) | 84 (77.1) | 21 (84.0) |  |
| **ABO-compatible** | | 126 (94.0) | 103 (94.5) | 23 (92.0) | 0.994 |
| **Ischemia time** |  |  |  |  |  |
|  | Cold ischemia time (hour) | 5.4 (0.7, 10.0) | 5.8 (0.7, 10.0) | 3.7 (0.4, 7.6) | 0.051 |
|  | Warm ischemia time (min) | 2.7 (2.0, 3.0) | 2.7 (2.0, 3.0) | 2.7 (2.0, 3.0) | 0.800 |
| **Acute rejection** |  | 0 (0) | 0 (0) | 0 (0) | / |
| **Delayed graft function** | | 12 (9.0) | 9 (8.3) | 3 (12.0) | 0.839 |
| **Intravenous immunogloblin** | | 0 (0) | 0 (0) | 0 (0) | / |
| **Induction treatment** | |  |  |  | 0.177 |
|  | Antithymocyte globulin | 12 (9.0) | 12 (11.0) | 0 (0.0) |  |
|  | Basiliximab | 122 (91.0) | 97 (89.0) | 25 (100.0) |  |
| **Initial immunosuppressive protocol** | |  |  |  | 0.600 |
|  | Tac+MPA+Pred | 108 (80.6) | 84 (77.1) | 24 (96.0) |  |
|  | CsA+MPA+Pred | 26 (19.4) | 25 (22.9) | 1 (4.0) |  |
| **Biochemistry** |  |  |  |  |  |
|  | Total bilirubin (μmol/L) | 9.4 (5.9, 11.3) | 9.7 (5.9, 12.0) | 8.0 (5.9, 9.8) | 0.491 |
|  | DB (μmol/L) | 2.5 (1.5, 2.8) | 2.6 (1.6, 3.0) | 2.0 (1.4, 2.5) | 0.087 |
|  | Total protein (g/L) | 65 ± 6 | 64 ± 6 | 65 (61, 69) | 0.606 |
|  | Albumin (g/L) | 44 ± 4 | 43 ± 5 | 45 ± 4 | 0.233 |
|  | Globulin (g/L) | 21 ± 4 | 21 ± 4 | 21 ± 4 | 0.572 |
|  | Alanine aminotransferase (U/L) | 21 (11, 24) | 21 (11, 26) | 21 (14, 24) | 0.795 |
|  | Aspartic aminotransferase (U/L) | 18 (13, 21) | 18 (13, 22) | 17 (13, 22) | 0.902 |
|  | Creatine (μmol/L) | 155 (104, 164) | 162 (108, 175) | 128 (98, 151) | 0.215 |
|  | Urea nitrogen (mmol/L) | 9.9 (6.6, 11.6) | 10.3 (6.9, 12,1) | 8.1 (6.0, 9.0) | 0.073 |
|  | Uric acid (μmol/L) | 370 ± 74 | 369 ± 73 | 378 ± 77 | 0.564 |
|  | eGFR (ml/min/1.73m^2^) | 54 ± 21 | 53 ± 22 | 57 (45, 69) | 0.464 |
| **Serum Protein electrophoresis** | |  |  |  |  |
|  | Albumin (%) | 63.1 ± 3.3 | 63.0 ± 3.2 | 63.8 (62.1, 65.9) | 0.274 |
|  | α1 (%) | 4.5 ± 0.8 | 4.5 ± 0.8 | 4.8 (4.3, 5.2) | 0.102 |
|  | α2 (%) | 9.4 ± 1.5 | 9.4 ± 1.4 | 9.4 (8.1, 10.7) | 0.895 |
|  | β (%) | 10.5 ± 1.2 | 10.5 ± 1.2 | 10.5 (9.7, 11.0) | 0.651 |
|  | γ (%) | 12.4 ± 2.7 | 12.6 ± 2.8 | 11.6 ± 2.4 | 0.126 |
| **Blood count** |  |  |  |  |  |
|  | RBC (×10^12^/L) | 3.84 ± 0.71 | 3.87 ± 0.74 | 3.74 (3.20, 4.02) | 0.356 |
|  | PLT/10 (×10^9^/L) | 20.6 ± 6.5 | 20.4 ± 6.5 | 21.2 (16.7, 25.5) | 0.710 |
|  | Neutrophil (×10^9^/L) | 5.3 (3.3, 6.6) | 5.5 (3.4, 7.1) | 4.3 (3.2, 5.4) | 0.044 |
|  | Lymphocy (×10^9^/L) | 1.6 (1.1, 2.0) | 1.6 (1.0, 2.0) | 1.7 (1.2, 2.0) | 0.454 |
|  | Monocyte (×10^9^/L) | 0.56 (0.39, 0.68) | 0.56 (0.39, 0.70) | 0.54 (0.50, 0.64) | 0.877 |
| **Urine protein** |  |  |  |  | 0.793 |
|  | （-） | 80 (69.7) | 64 (58.7) | 16 (64.0) |  |
|  | （±） | 26 (19.4) | 20 (18.3) | 6 (24.0) |  |
|  | （+~++） | 19 (14.2) | 17 (15.6) | 2 (8.0) |  |
|  | （++~+++） | 8 (6.0) | 7 (6.4) | 1 (4.0) |  |
|  | （+++~++++） | 1 (0.7) | 1 (0.9) | / |  |

Note: For continuous variables, the fitting normal distribution ones are expressed as the mean ± SD, otherwise expressed as the median with interquartile ranges when data. For categorical data, the proportions and frequencies are calculated. Continuous and categorical variables were compared using independent t test or nonparametric and χ2 tests. DB, direct bilirubin; Tac, tacrolimus; CsA, cyclosporine A; MPA, mycophenolic acid; Pre, prednisone; eGFR, estimate glomerular filtration rate; RBC, red blood cells.
